# Supplementary material for: Influence of Socioeconomic Status on SARS-CoV-2 Infection in Spanish Pregnant Women. The MOACC-19 Cohort
Source: Int J Environ Res Public Health. 2021 May 12;18(10):5133. doi: 10.3390/ijerph18105133 (PMC8150608; doi:10.3390/ijerph18105133)

Title: Influence of socioeconomic status on SARS-CoV-2 infection in Spanish pregnant women. The MOACC-19 cohort

Authors: Javier Llorca[1,2], Carolina Lechosa-Muñiz[1,3,] Lorena Lasarte-Oria[3], Rocío Cuesta-González[3], Marcos López-Hoyos[1,3,4], Pilar Gortázar[1,3], Inés Gómez-Acebo[1,2,4], Trinidad Dierssen-Sotos[1,2,4], María J. Cabero-Pérez[1,2] and the MOACC-19 group

Affiliations:

1: Universidad de Cantabria, Santander, Spain.

2: CIBER Epidemiología y Salud Pública, Madrid, Spain.

3: Hospital Universitario Marqués de Valdecilla, Santander, Spain

4: IDIVAL, Santander, Spain

Members of the MOACC-19 group:

Jéssica Alonso-Molero[1,4], Bárbara Arozamena[3], Laura Conde-Gil[3], Elsa Cornejo del Río[3], María Fernández-Ortiz[3], Pelayo Frank de Zulueta[3], Yolanda Jubete[1,3], Coral Llano-Ruiz[3], Lorena Lasarte-Oria[3], Sonia López-Gómez[3], Sonia Mateo-Sota[3], Victoria Orallo[3], Rosa Pardo[3], Daniel Pérez González[3], María Sáez de Adana Herrero[3].

Correspondence:

Javier Llorca

Universidad de Cantabria

Facultad de Medicina

Avda. Herrera Oria s/n

39011 Santander

SPAIN

Phone # 34-942 201 993

E-mail: javier.llorca@unican.es

Supplementary Figures

Figure S1. Association between housing surface (continous variable) and infection by SARS-CoV-2 by cubic spline regression. Solid black line indicates the central estimation, dashed lines are limits of 95% confidence interval. Orange line indicates the distribution of housing surface (right Y axis).

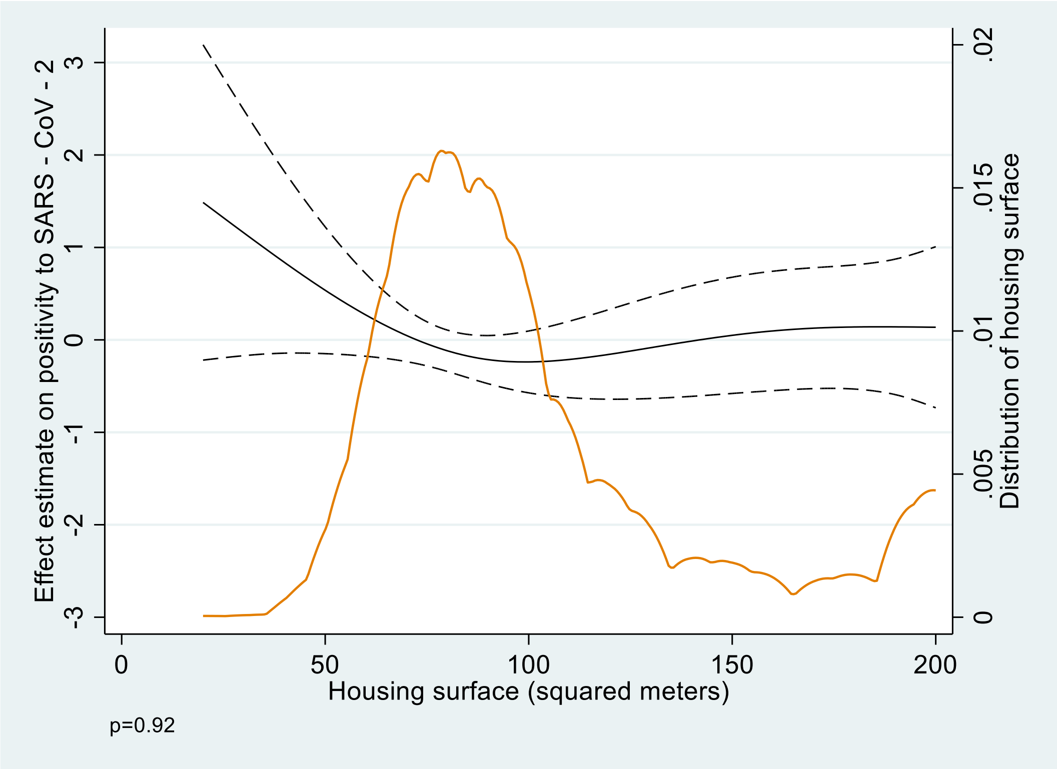

Figure S2. Association between year of building (continous variable) and infection by SARS-CoV-2 by cubic spline regression. Solid black line indicates the central estimation, dashed lines are limits of 95% confidence interval. Bars indicate the distribution of year of building (right Y axis).

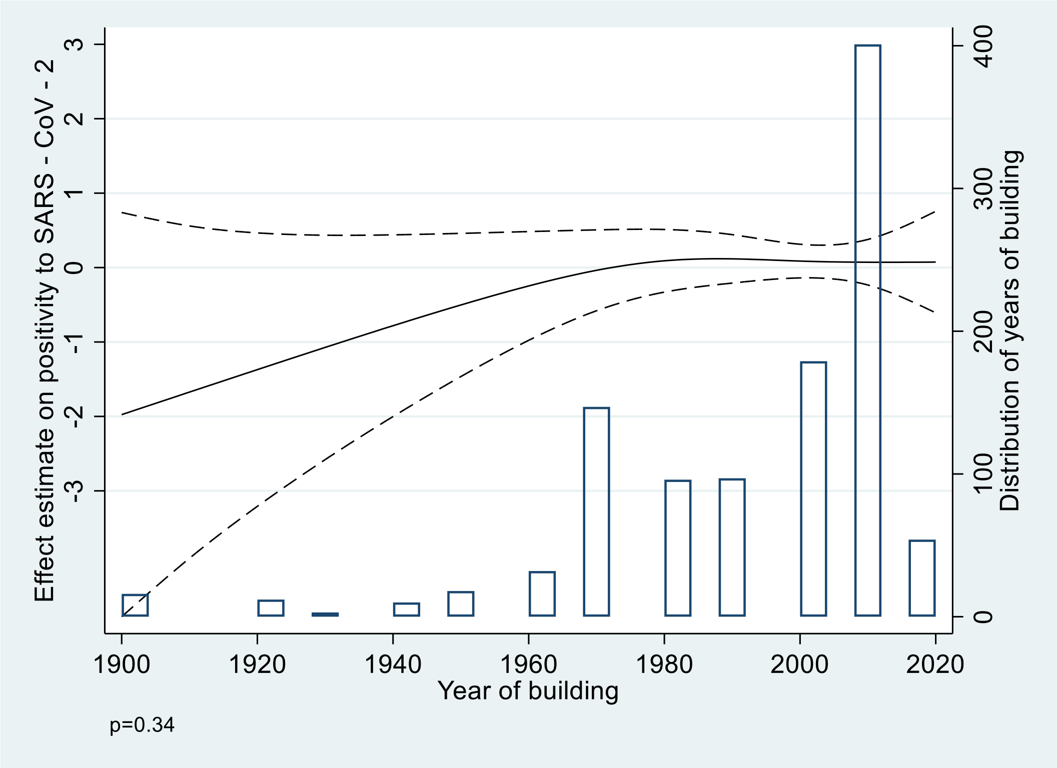

Figure S3. Association between number of bathrooms and infection by SARS-CoV-2 by cubic spline regression. Solid black line indicates the central estimation, dashed lines are limits of 95% confidence interval. Bars indicate the distribution of the number of bathrooms (right Y axis).

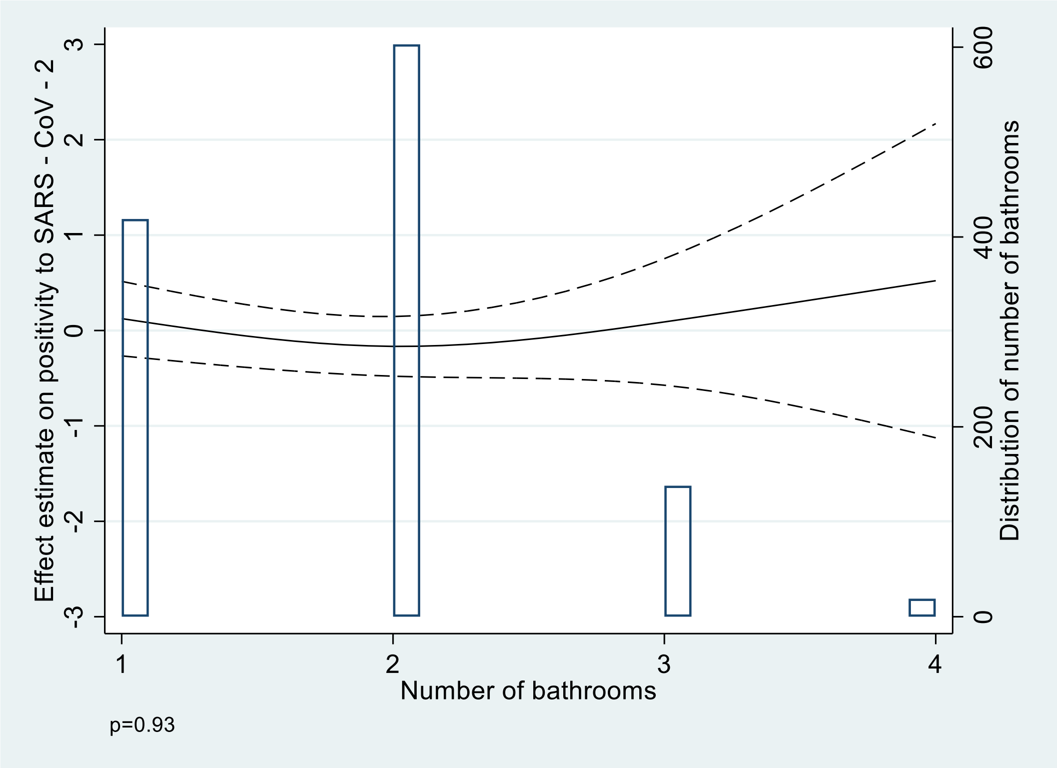

Figure S4. Association between number of rooms and infection by SARS-CoV-2 by cubic spline regression. Solid black line indicates the central estimation, dashed lines are limits of 95% confidence interval. Bars indicate the distribution of the number of rooms (right Y axis).

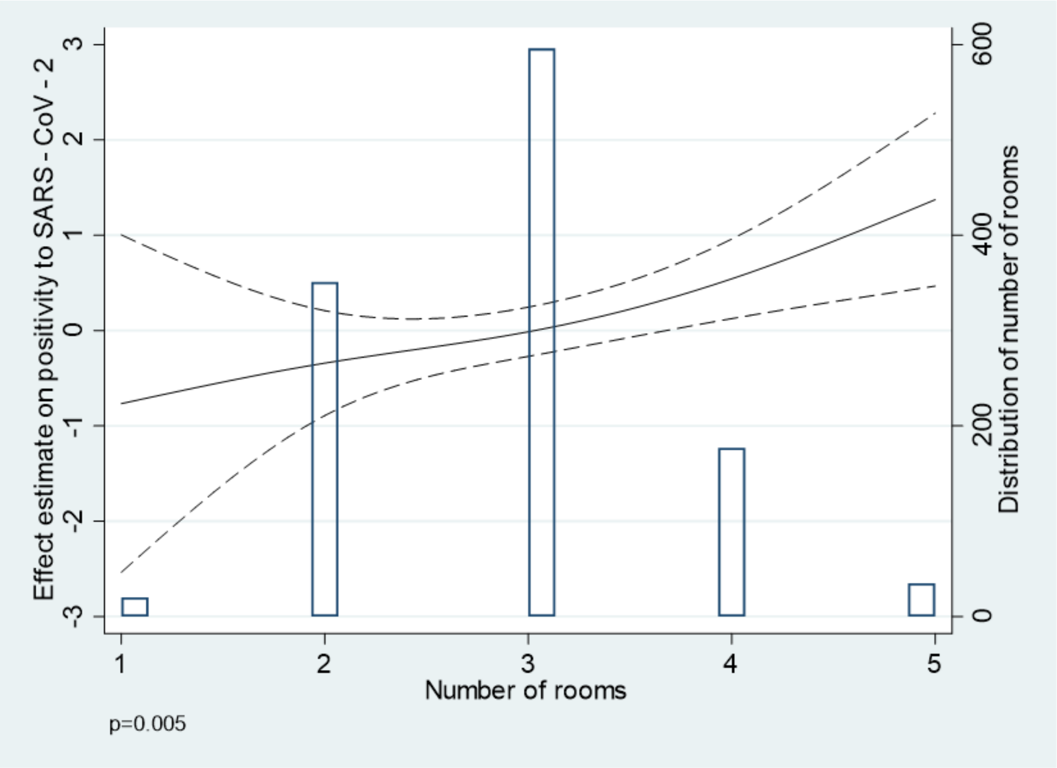

Figure S5. Association between number of people in the household and infection by SARS-CoV-2 by cubic spline regression. Solid black line indicates the central estimation, dashed lines are limits of 95% confidence interval. Bar indicate the distribution of number of people in the household (right Y axis).

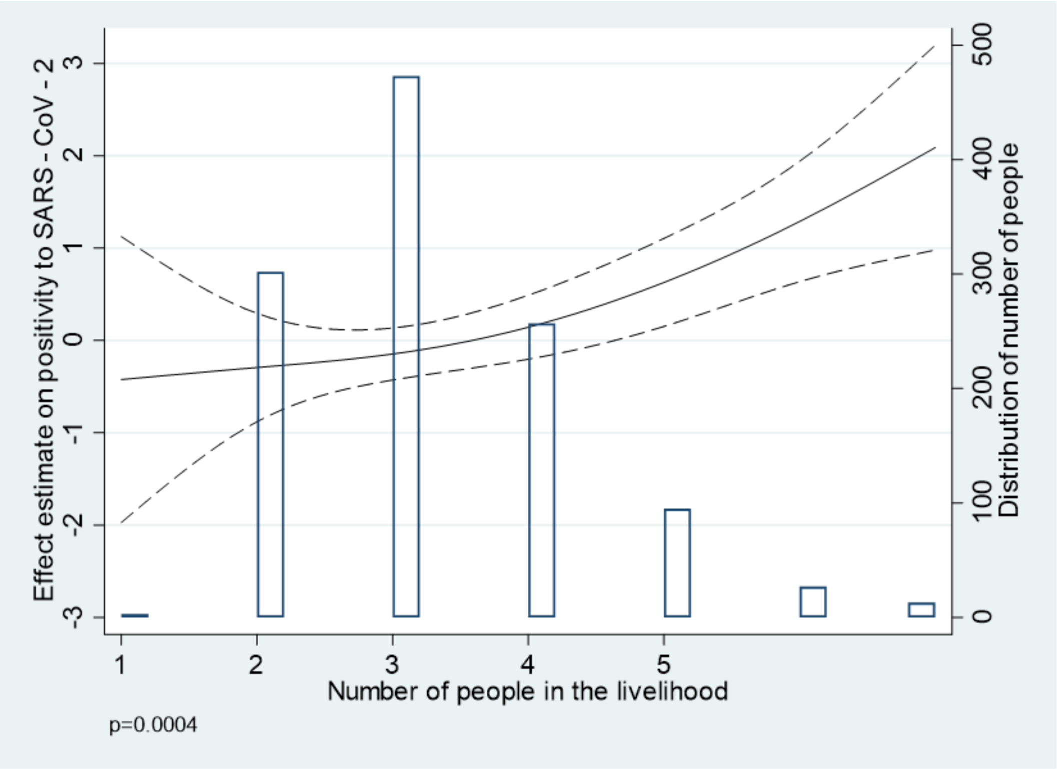

Figure S6. Association between housing surface per person (continuous variable) and infection by SARS-CoV-2 by cubic spline regression. Solid black line indicates the central estimation, dashed lines are limits of 95% confidence interval. Blue line indicates the distribution of housing surface per person (right Y axis).

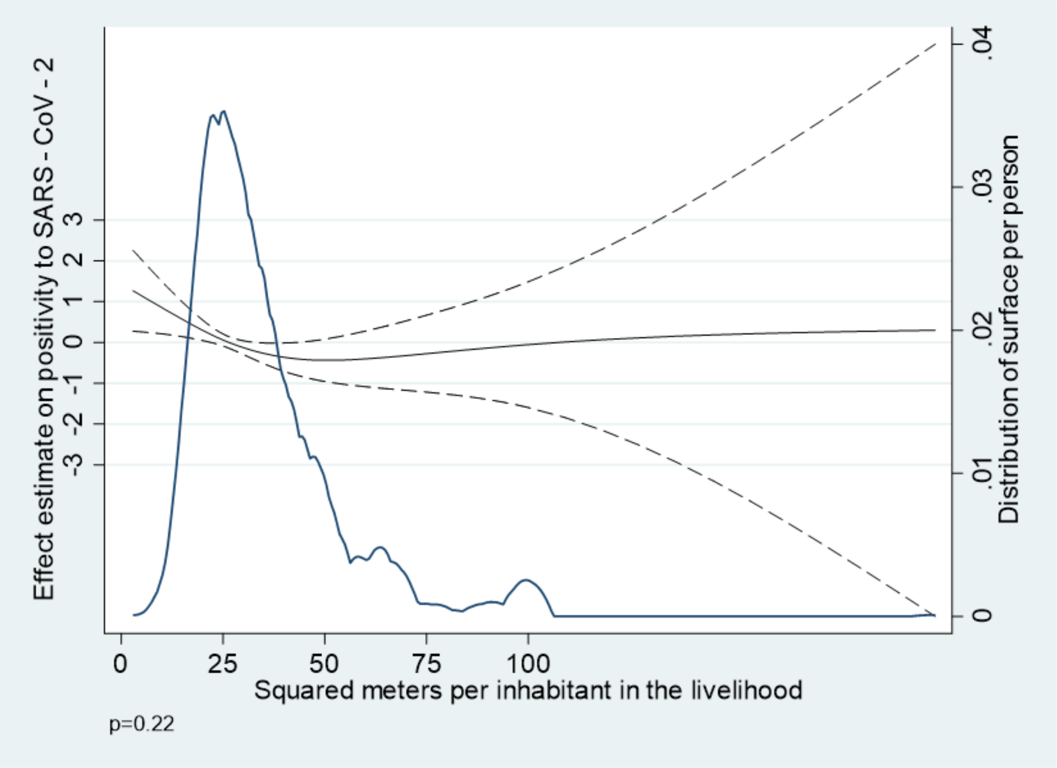

Figure S7. Association between housing score and infection by SARS-CoV-2 by cubic spline regression. Solid black line indicates the central estimation, dashed lines are limits of 95% confidence interval. Bars indicate the distribution of housing score (right Y axis).

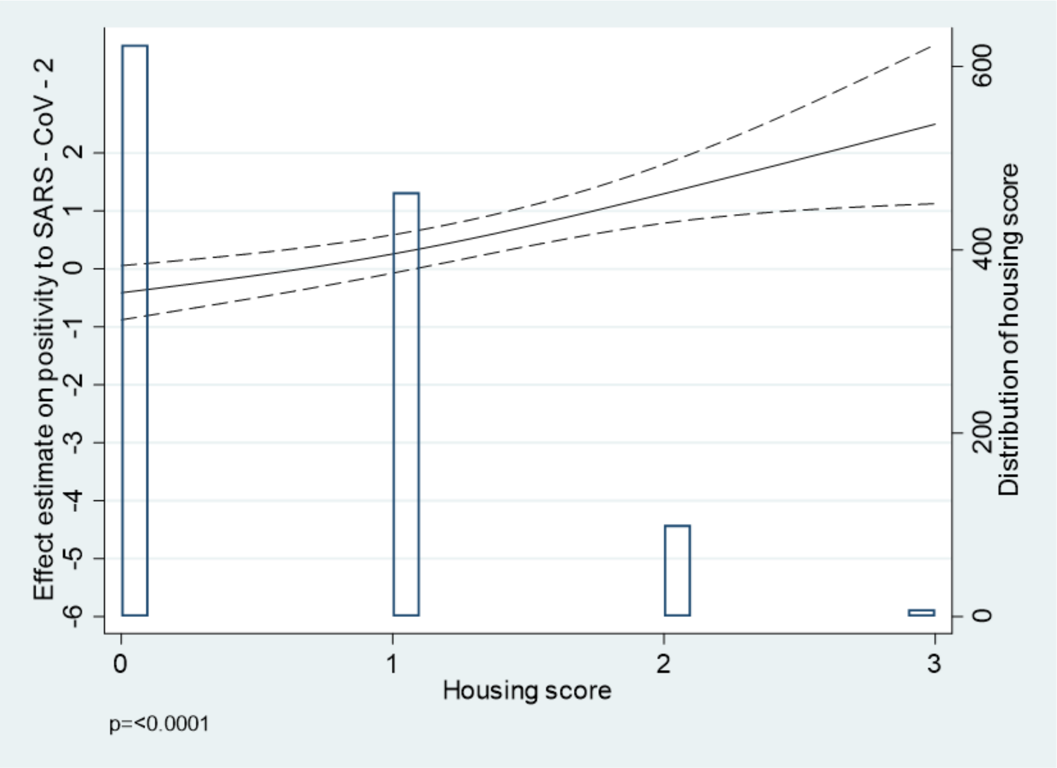

Supplement: Supplementary file 1 [file ijerph-18-05133-s001.zip › ijerph-1198498-supplementary.pdf]
